# Interaction between stress hormones and phagocytic cells and its effect on the health status of dairy cows: A review

**DOI:** 10.14202/vetworld.2020.1837-1848

**Published:** 2020-09-10

**Authors:** Mohanned Naif Alhussien, Ajay Kumar Dang

**Affiliations:** Lactation and Immuno-Physiology Laboratory, ICAR-National Dairy Research Institute, Karnal, Haryana, India

**Keywords:** dairy cattle, inflammatory diseases, leukocyte trafficking, macrophages, neutrophils

## Abstract

Dairy cows are exposed to various stressors during their production cycle that makes them more susceptible to various diseases. Phagocytes (neutrophils and macrophages) are important soldiers of the innate immune system. Neutrophils are the first responders to an inflammatory response and stress and kill pathogens by generating reactive oxygen species and by the release of various antimicrobial peptides, enzymes, neutrophil extracellular trap formation, etc. Macrophages, the other phagocytes, are also the cleanup crew for the innate immune system that removes debris, pathogens, and dead neutrophils later on after an inflammatory response. The neuroendocrine system along with phagocytes exhibits an immunomodulatory potential during stressful conditions. Neuroendocrine system directly affects the activity of phagocytes by communicating bidirectionally through shared receptors and messenger molecules such as hormones, neurotransmitters, or cytokines. Different immune cells may show variable responses to each hormone. Short time exposure to stress can be beneficial, but repeated or extended exposure to stress may be detrimental to the overall health and well-being of an animal. Although some stresses associated with farming practices in dairy cows are unavoidable, better understanding of the interactions occurring between various stress hormones and phagocytic cells can help to reduce stress, improve productivity and animal welfare. This review highlights the role played by various stress hormones in modulating phagocytic cell performance of dairy cattle under inflammatory conditions.

## Introduction

High-producing dairy cows have high metabolic demands around calving and during early lactation where the chances of developing health disorders are quite high [1-3]. This condition gets further aggravated when accompanied by extreme environmental conditions which provide an optimum condition for microbial growth and multiplications [4,5]. Adequate sensation and timely response of animals to these changes by neuroendocrine and immune system enable them to maintain their health and reproduction [6,7]. Animals experience various infections throughout their lifespans and they respond to these attacking pathogens by activating their highly sensitive receptor-based perception systems activating antimicrobial signaling cascades. For this, both polymorphonuclear leukocytes (PMNs) and macrophages (collectively known as phagocytic cells) have a highly sophisticated defense system, which enables them to precisely recognize pathogen molecules and clear them from the system [8-12]. The phagocytes (phagein means to eat and cyte stands for the cell) protect the body by ingesting harmful foreign particles and have long been considered as essential cells for defense mechanism against invading pathogens. One hundred and fifteen years ago, Elie Metchnikoff discovered phagocytosis and suggested that the key to effective immunity was to “activate the phagocytes [13].” The activity of phagocytic cells, expression of surface adhesion molecules, as well as stress hormone receptors get modulated in response to environmental stress [14-16], physiological stress such as calving and early lactation [14,17], and pathological stress such as mammary and uterine infections [18-23].

Stress is detrimental to health, and different stress hormones released during unfavorable conditions have multiple and different effects on the functionality and large-scale redistribution of immune cells [15,16,24,25]. These hormones are cortisol, catecholamine, growth hormones (GH), prolactin (PRL), histamine, apelin, etc. Unlike glandular endocrine cells, phagocytic cells are considered to be both poly producers and poly receivers of hormones [26,27]. Since they are mobile cells, they can transport the stored hormone to the inflamed tissue contributing to the local synthesis and secretion of various hormones [28-33]. Although efforts have been made in the past to unravel the role of various hormones on phagocytes, understanding the molecular events by which stress hormones affect innate immunity is still unknown. Therefore, this review is an effort to address the latest breakthrough discovery related to the role of stress hormones in the modulation of phagocytic cells activity of dairy cows. Ultimately, a better understanding of the interactions between the stress hormones and the phagocytic cells can open up new therapeutic approaches and improve the well-being and sustainability of our dairy herds.

## Phagocytes Activation, Migration, and their Mode of Action

**Figure-1:**
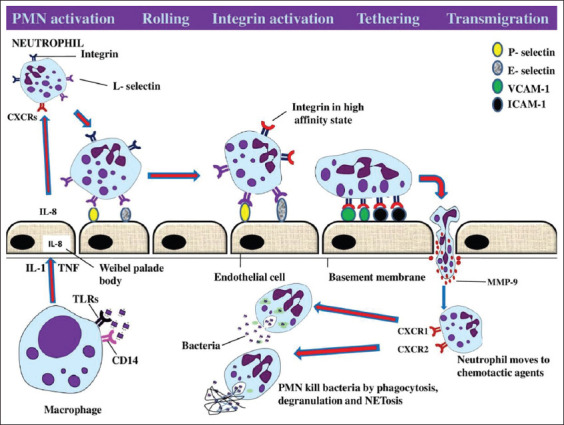
Diagrammatic representation of phagocytic cells activation and subsequent migration at the site of infection. Initially, macrophages in the tissue detect the presence of pathogens through CD-14 and toll-like receptors and secrete inflammatory cytokines such as IL-1 and tumor necrosis factor which further stimulate the release of interleukin (IL-8) from Weibel-Palade bodies as well as expression of both P-selectin and E-selectin on the surface of endothelial cells (ECs). IL-8 binds to the chemokine receptors (CXCR1 and CXCR2) on the surface of polymorphonuclear leukocytes (PMNs) which causes PMNs activation. Activated neutrophils use L-selectin and integrin for slowing down and tethering to the ECs, respectively. Matrix metalloproteinases help in transmigration and chemokine receptors help PMNs to move toward the chemotactic gradients and perform their functions by various mechanisms. (Source: Mohanned Naif Alhussien and Ajay Kumar Dang).

The main phagocytic cells are macrophages in the tissues and PMNs in the bloodstream. Macrophages activation and PMNs migration process can be split up into several stages. It starts when activated macrophages release pro-inflammatory cytokines essentials for PMNs activation in the blood vessel, subsequent migration of PMNs to the inflamed site, and clearance of the pathogens [12]. Under normal physiological conditions, neutrophils circulate in the blood and survey for microbial-associated molecular patterns. Neutrophils are short-lived and are replaced every 24 h. During the periods of stress, phagocytes get activated and express their secretory and cell membrane activity [11,34]. Many receptors, adhesion molecules, and cytokines are involved in the activation, migration, and fighting ability of phagocytes in response to an inflammatory process (Figure-1). The presence of a pathogen stimulates an inflammatory response in the host body through bacteria-derived inflammatory signals such as lipopolysaccharide (LPS), host-derived chemoattractants, and cytokines such as tumor necrosis factor (TNF)-α and interleukins [35]. These signals stimulate the endothelial cells to express adhesion molecules on the luminal surface, that is, the P-selectin (CD62P), E-selectin (CD62E), and endothelial intercellular adhesion molecules (ICAM-1 and ICAM-2). Thereafter, these adhesion molecules then get attached to the β2 integrin (CD11b/CD18) and L-selectin (CD62L) of neutrophils leading to tethering followed by rolling of neutrophils along the wall of the blood vessel [12,35].

Binding of neutrophils to the endothelium forms a foundation for the beginning of transmigratory activity of neutrophils through the endothelial lining through interaction with its surface receptors (integrins) [36]. Eventually, matrix metalloproteinases, also known as collagenase, are released which help neutrophils to dissolve and penetrate the basement membrane and reach the site of inflammation. Moreover, the chemotactic gradient created by bacteria as well as host-derived chemokines facilitates the migration of neutrophils toward the site of inflammation. Phagocytes are also equipped with pattern recognition receptors which are capable of recognizing specific non-self-patterns present on foreign materials like the family of toll-like receptors (TLRs) [37]. Binding of phagocytic surface receptors to the complement components and immunoglobulins results in further cell activation, and increased oxygen consumption and hexose monophosphate activity in a process called the “respiratory burst [35].” The intracellular granules of phagocytes contain different enzymes, that is, defensins, myeloperoxidase, elastase, and other enzymes which can efficiently kill a variety of invading organisms [12,35]. In addition, neutrophils can also trap and kill any foreign materials by releasing thread-like structures called neutrophil extracellular traps (NETs) [38,39]. Milk neutrophils have been reported to use NETs as a strategy in fighting against mastitis pathogens during mammary infections [40]. Once the neutrophils have fulfilled their physiological functions in the tissue, they undergo apoptosis and get cleared by the macrophages [35].

## Neuroendocrine Responses to Stress

**Figure-2:**
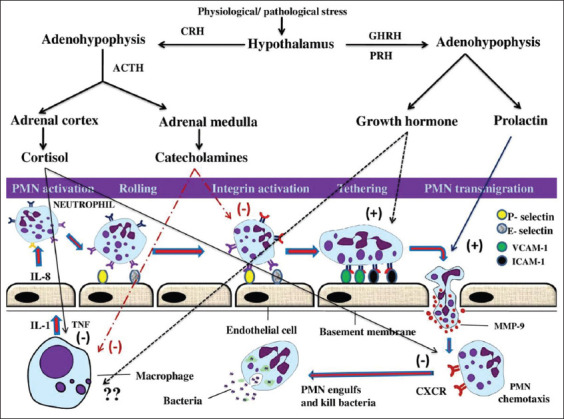
Stress modulation of the hormonal profile by the central nervous system. On experiencing an adverse stimulus (stressor), the hypothalamic–pituitary–adrenal axis and the sympathetic nervous system are activated resulting in the release of glucocorticoids and catecholamines, which can modulate and suppress various receptors and adhesion molecules involved in the activity of phagocytic cells. Furthermore, the pituitary hormones (prolactin and growth hormone) are also released which, however, enhance phagocytic cells activity. GHRH: Growth hormone-releasing hormone; PRH: Prolactin-releasing hormone; CRH: Corticotropin-releasing hormone; ACTH: Adrenocorticotropic hormone. Negative (−) sign indicates an inhibitory effect, while positive (+) sign indicates a stimulatory effect. (Source: Mohanned Naif Alhussien and Ajay Kumar Dang).

Stress in any form, physiological or pathological, causes the release of neuroendocrine signals from the brain. The main two neuroendocrine pathways activated in response to stress and control the immune system are the hypothalamic–pituitary–adrenal (HPA) axis and sympathetic nervous system (SNS) [28,32]. Stimulation of these two systems results in the release of glucocorticoids and catecholamines (epinephrine and norepinephrine), respectively (Figure-2). However, other neuroendocrine hormones (PRL, GH, histamine, and apelin) are also released following stress and involved in the regulation of phagocytic cells activity (Figure-2). Stress hormones serve as beacons that make the immune system ready for potential challenges perceived by the brain. Nevertheless, the homeostasis of this system may break down if the enhanced immune response is dysregulated after long activation, as seen during chronic stress [32,33,41,42].

## Role of HPA Axis in Innate Immunity

**Figure-3:**
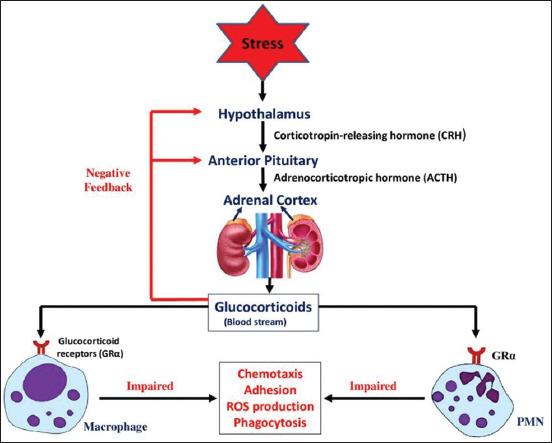
Diagrammatic representation of activation of the hypothalamic–pituitary–adrenal axis during stress/inflammation and its subsequent effects on the activity of phagocytes. (Source: Mohanned Naif Alhussien and Ajay Kumar Dang).

Hypothalamus of an animal receives and monitors information from the environment and coordinates it through various nerves and hormones. For optimum immunological response, a complex interaction occurs between the HPA axis and various constituents of the immune system. The brain regulates the immune system through activation of the HPA axis. On stimulation of this axis, corticotropin-releasing hormone is secreted from the paraventricular nucleus of the hypothalamus (Figure-3). This causes secretion of adrenocorticotropic hormone from the anterior pituitary into the circulation which further induces the adrenal glands to synthesize and secrete glucocorticoids [43]. Cortisol is a well-known marker for physiological stress and cattle with higher temperament scores exhibit greater cortisol concentration in their blood [44]. Cortisol has been reported as a stress marker in the milk of dairy cows [16,45]. Brown and Vosloo [46] demonstrated that intensification of dairy farming practices has increased the plasma cortisol levels in dairy animals. Although some of these practices are unavoidable, improving farming methods can lower the cortisol levels, increase the welfare and efficiency of cattle production.

Both PMNs and macrophages exhibit relatively abundant levels of glucocorticoid receptors in their cytoplasm and, through these receptors, the glucocorticoids tend to exert multiple effects on these cells (Figure-3). Studies have demonstrated that phagocytic activity of neutrophils gets diminished with a concomitant increase of plasma cortisol in the blood and milk of both parturient and mastitis cows [17,47,48]. A dramatic impairment in random migration, adhesion, and reactive oxygen species (ROS) production of blood PMNs has been observed during the 1^st^ week after parturition and during mammary infection in association with plasma cortisol elevation [41].

A linear relationship (positively correlated) was observed between cortisol concentration and expression of both chemokine receptors (CXCR1 and CXCR2) on PMNs of parturient and mastitis [17]. As discussed earlier, effective recruitment of phagocytes to the site of infection requires adhesion molecules, including the selectin and β_2_ integrin families. Selectin (CD-62L) slows down the phagocytic cells and allows them to roll along vascular endothelial cells, while integrin (CD-11b) mediates tethering of PMNs to the endothelial cells. A linear relationship was also observed between cortisol concentration and the expression of integrin on both blood and milk PMNs during stressful conditions (mastitis and parturition), indicating more transmigration efficiency of neutrophils to the site of inflammation under such circumstances [17,48]. An increase in the plasma cortisol hormone has been recorded during extreme weather conditions [49,50], which, in turn, caused immune disturbance, higher inflammatory response and decreases phagocytic activity of neutrophils during summer season [14-16,44]. Even the ability of neutrophils to detect invading pathogens is decreased as reflected by the lower expression of the TLRs on the surface of neutrophils during this period. Alhussien and Dang [16] observed higher milk cortisol concentrations in association with increased expression of heat shock proteins and CD molecules in neutrophils during heat stress in Indian native breeds of cattle.

Macrophages, through antigen presentation, act as a bridge linking innate and acquired immunity. They activate both T and B lymphocyte, enabling them to secrete cytokines, exert cytotoxic, suppressor, and memory functions [51]. As observed in PMNs, the activity of the macrophages also gets suppressed under the influence of glucocorticoids. Even administration of dexamethasone (a potent glucocorticoid) to dairy calves reduces the mRNA expression of TLRs and acute-phase cytokine on macrophages [52]. Macrophages are the prominent leukocyte in the milk of healthy animals but get highly outnumbered by PMN during mammary inflammation [48]. Mukherjee *et al*. [53] studied the effect of plasma cortisol on the activity of milk macrophage during different stages of lactation cycle. The highest phagocytic index (PI) of milk phagocytes was observed at calving and early and mid-stage of lactation which was positively correlated with plasma cortisol levels. However, during the late stage of lactation, an inverse relationship was observed between PI and plasma cortisol levels.

Recently, the anti-inflammatory effects of cortisol and the molecular mechanisms that mediate its action have been reported in ruminants. Dong *et al*. [42] demonstrated that high cortisol concentrations attenuate LPS-induced inflammatory responses in the RAW264.7 macrophage cell line by regulating the nuclear factor-kappa B (NF-κB) and mitogen-activated protein kinases (MAPK) signaling pathways. Cui *et al*. [54] demonstrated a two-side effect of cortisol in bovine endometrial epithelial cells (BEECs) stimulated with LPS, heat-killed *Escherichia coli* or live *E. coli*. They found that cortisol had anti-inflammatory effects on LPS- or heat-killed *E. coli*-stimulated BEEC, but displayed pro-inflammatory action on live *E. coli*-induced BEEC. Cui *et al*. [55] reported that cortisol impairs *E. coli*-induced endometrial inflammatory response through NF-κB and MAPK pathways in postpartum goats.

### The Role of SNS During Stress/Inflammation

The SNS is mainly involved in the stimulation of fight or flight response to maintain homeostasis in the body. Activation of the SNS results in secretion of acetylcholine from the pre-ganglionic sympathetic fibers in the adrenal medulla. This induces the secretion of epinephrine into the systemic blood supply. Norepinephrine (a member of catecholamine family) is released from the nerve terminals in the vicinity of immune cells. Catecholamine concentrations are elevated in the blood of dairy cattle during stressful conditions [56]. The majority of immune cells (PMNs, macrophages, T and B lymphocytes, and NK cells) express β2-adrenergic receptors on their surface. Excitation of the SNS causes the release of catecholamine into the circulation, which exerts their effects on immune cells that express adrenergic receptors on their surface [57]. These effects cause changes in immune cellular trafficking, proliferation, antibody production, and cytokine secretion and activity [58,59].

In bovine PMNs, the β2-adrenergic receptor signaling pathway is similar to that described in other species as well as the potential for an inflammatory stimulus to alter its function [60]. In physiologically stressed cows, high levels of blood catecholamine were involved in the impairment of CD-11b-mediated PMNs functions [61]. Moreover, epinephrine has been reported to modulate the trafficking of phagocytes, regulate cytokine secretion, and change host-pathogen interactions by altering microbial growth [32,62-64].

Macrophages not only respond to catecholamines, but they can also produce them as well. Activation of both (α_1_-AR and β_2_-AR) suppresses the production of ROS in bovine alveolar macrophage [65]. During periods of immune system activation, bovine macrophages are involved in modulating catecholamine secretion from the adrenal chromaffin cell of adrenal medulla through releasing prostaglandin E2 and other chemical signals [66]. Glucocorticoid and catecholamine have combined effects on monocyte/macrophage and dendritic cells to inhibit the secretion of various pro-inflammatory cytokines and the acute-phase proteins while promoting the production of T-helper 2 humoral related cytokines [67,68]. Reiske *et al*. [59] studied the effects of *in vitro* treatment of porcine peripheral blood mononuclear cells with cortisol, adrenaline, or noradrenaline at increasing concentrations resembling low to high stress conditions. They reported that cortisol caused a decrease in the number of TNF-α-producing cells and the mitogen-induced lymphocyte proliferation. However, catecholamines increased proliferation while exerting suppressive effects on the number of cytokine producers.

## Lactogenic Hormones During Infection and Immunity

The lactogenic hormones are secreted by the anterior pituitary and stimulate mammogenesis and lactogenesis in the females. Both PRL and GH are necessary for the transition of the mammary gland from a proliferative to a lactating state. Indeed, they have been associated with structural and functional changes during the dry period and onset of lactation (period of mammary stress) in dairy cattle. Interestingly, these changes and lactogenic hormones elevation have been related to the periods of the highest incidence of infections [69,70].

### PRL

PRL, a protein hormone is synthesized and secreted from particular cells of the anterior pituitary gland, named lactotrophs. Several cells of the immune system such as macrophages, NK cells, and lymphocytes are considered as important sources for PRL synthesis [26,71,72]. In addition to its role in lactation, PRL has been shown to have significant involvement in immune functions, playing an important role in signaling between immune and neuroendocrine systems [26,27]. During infection and inflammation, PRL plays a critical role in maintaining the immune system homeostasis [26].

PRL triggers the pro-inflammatory immune response and PRL within physiologically achievable concentrations mediates PMNs migration of dairy cows [73]. Moreover, PRL induces phagocytosis of different microorganisms in both phagocytic (macrophages, PMNs) and non-phagocytic (epithelial) cells. In response, these cells produce pro-inflammatory cytokines and nitric oxide (NO), necessary for leukocyte recruitment to the site of infection. ROS and NO production in mice treated with PRL have reduced mortality and increased the phagocytic activity of macrophages in them [74]. However, PRL does not modify NO production in bovine macrophages infected with *Mycobacterium avium* subspecies *paratuberculosis* (*M. avium* ss. *paratuberculosis*). PRL can suppress the ability of bovine monocytes to control the intracellular growth of *M. avium* ss *. paratuberculosis* *in vitro*. Moreover, fluctuations in the levels of GH and PRL during parturition and lactation might make bovine mononuclear phagocytes more susceptible for the intracellular growth of *M. avium* ss. This phenomenon might be the main cause for the incidence of Johne’s disease in dairy cattle [75].

Gutiérrez-Barroso *et al*. [76] have demonstrated that PRL *in vitro* together with *Staphylococcus aureus* downregulated mRNA expression of β-defensin and interleukin-1β as well as NO production in bovine mammary epithelial cells. These observations imply that PRL can inhibit host innate immune response elements during infection. Boutet *et al*. [73] reported no difference in the circulating levels of plasma PRL between healthy and mastitis-affected cows. However, milk PRL concentration increased in chronic mastitis-affected quarters and had a positive correlation with somatic cell count as well as the number of PMNs migrated to the milk. PRL is also reported to stimulate an inflammatory response in bovine mammary epithelial cells through NF-kB activation as well as cell adhesion expression, which results in more PMNs trafficking to the infected site during chronic mastitis [73].

Dairy steers during long-day photoperiods had higher levels of circulating PRL but reduced expression of PRL receptor on peripheral blood mononuclear cells compared to those exposed to short-day photoperiod [77]. Neutrophils isolated from cows assigned to a short-day photoperiod during the dry period had higher cellular trafficking toward various chemoattractants, while their lymphocytes showed enhanced cellular proliferation [78]. The highest phagocytic activity and PI of milk PMNs have been observed during mid-lactation while the lowest phagocytic activity and index seen during early lactation [14]. Similarly, lactation stage-specific effect of PRL on the mammary immunity has also been observed [53]. This is because PRL and other pituitary hormones are suggested to act as stress adaptation molecules essential for maintaining the homeostasis of mammary gland immunity throughout the lactation cycle. The inhibition of PRL has been reported as a promising strategy for reducing metabolic and acute nutritional stress during the postpartum period without compromising the overall productivity of dairy animals. Moreover, an improvement in some aspects of immune system has been associated with the inhibition of PRL during this period [70,79].

Research carried out on PRL over the recent years indicates that it functions as a survival factor, promotes proliferation, and inhibits apoptosis. These critical functions are important to maintain the desired number of immune cells in physiological conditions and also to enhance immune tolerance. Abnormal synthesis and secretion of PRL can disturb homeostasis of the immune system and subsequently promote autoreactivity or aggravation of the clinical condition in autoimmune diseases.

### GH

**Figure-4:**
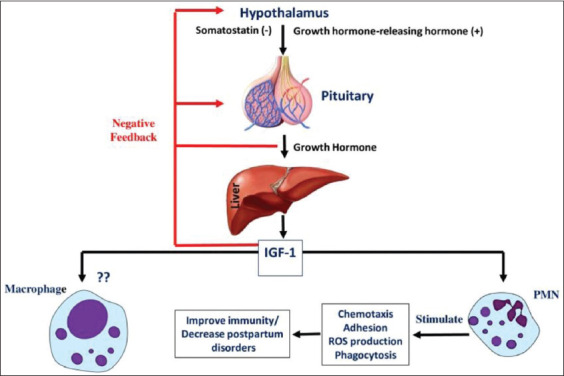
Diagrammatic representation of neuroendocrine activation of the growth hormone axis and its subsequent effects on the activity of phagocytes. IGF-1: Insulin-like growth factor 1. Negative (−) sign indicates an inhibitory effect while positive (+) sign indicates a stimulatory effect. (Source: Mohanned Naif Alhussien and Ajay Kumar Dang).

GH, also known as somatotropin, is another peptide hormone secreted by somatotropic cells of the anterior pituitary (Figure-4). Secretion of GH in the pituitary is regulated by two peptides, GH-releasing hormone and GH-inhibiting hormone (somatostatin) secreted from the hypothalamus (Figure-4). GH secretion from the pituitary is predominantly determined by the balance of these two peptides, which, in turn, is affected by the physiological/pathological status of the animal. Apart from the development of the mammary gland (ductal morphogenesis), milk synthesis, and metabolic regulation of periparturient dairy cows, it can also exert a direct effect on all major immune cell types and participate in the development and maintenance of both innate and adaptive immunity [80]. This hormone stimulates the production of insulin-like growth factor (IGF-1), which also has been found to be involved in the development of immune responses such as T-cell proliferation and activation, chemotaxis, apoptosis, and natural killer cell cytotoxicity [81,82].

Somatotropin and IGF-1 are thought to be involved in several immune functions [83], and actions of bovine somatotropin (bST) can either be direct or indirectly mediated through IGF-1. An increased number of circulating leukocytes, band PMNs, and respiratory burst activity in cows treated with bST after calving have been reported [84]. No effect was observed *in vitro* as well as *in vivo* on phagocytosis and killing of *E. coli* by circulating PMN or on cytochrome c reduction [83]. However, an increase in the PMNs production of ROS after *in vivo* administration of bST has been reported in healthy cows [85]. bST does not affect chemotaxis and the expression of adhesion receptors of circulating PMNs during *Streptococcus uberis* mastitis, which is in contrast with coliform mastitis. Nevertheless, the absence of such effects is not essential for the outcome of *S. uberis* infection because the removal of *S. uberis* from the mammary gland is mainly dependent on macrophages rather than PMNs [86].

The activity of phagocytic cells from cows diagnosed with metritis was reduced during the prepartum period [87]. The ability of PMNs to perform phagocytosis and oxidative burst increased during the prepartum period in recombinant bST (rbST125) treated cows [88]. Treatment of cows with 142.8 mg of rbST during the transition period did not affect metritis incidence but reduced the overall incidence of periparturient diseases [89]. However, the incidence of metritis and retained fetal membranes was decreased considerably in rbST125 treated cows, which may be due to the enhancement of PMNs and macrophages activity by rbST [88,90].

In the human model, GH reported modulating the acute-phase response by increasing IGF-1 and decreasing interleukin-1 expression, which subsequently causes a decrease in type 1 acute-phase proteins and increases in the constitutive hepatic proteins [91]. Metritis cows treated with rbST had a considerable decrease in the TNF-α concentrations [88]. The great role played by GH in enhancing immunity by acting directly or indirectly on immune cells, suggests that rbST treatment during transition period might be quite beneficial. Administration of somatotropin in transition cows could be used as a prepartum strategy to increase IGF-1 concentrations, improve immune function, and decrease postpartum disorders.

## Histamine

Histamine is an organic nitrogenous compound produced by histaminergic neurons located in the hypothalamus or released by mast cells, basophils, platelets, and enterochromaffin-like cells. Histamine mediates its effects by four types of receptors (H1, H2, H3, and H4), which belong to the GPCRs family. Depending on the histaminergic receptor stimulated, histamine can either suppress or stimulate inflammatory reactions [92].

Many reports have found an essential and direct role for histamine in the regulation of PMNs dominant inflammatory reactions [93,94]. H4R can be strongly used as a novel target for the pharmacological mod*u*lation of histamine-transferred immune signals [95]. Despite the fact that histamine is predominantly preformed and stored in mast cells and basophils, recent evidence suggested that PMNs can also synthesis and release histamine during the inflammatory process. Smuda *et al*. [93] reported that bone marrow-derived PMNs when stimulated with a range of TLR agonists, secreted histamine in response to LPSs. LPS-stimulated histamine release was also enhanced by coculture with granulocyte macrophage colony-stimulating factor. In equine, it has been observed that high concentrations of histamine *in vitro* stimulated PMNs to produce ROS through histamine H1 receptors and the nicotinamide adenine dinucleotide phosphate oxidase pathway [96].

In bovines, Anderson *et al*. [97] identified the presence of histamine receptor on the bovine leukocytes, mainly the lymphocytes. The *in vitro* effects of histamine on the chemiluminescence response of bovine PMNs were determined [98] and the addition of histamine was found to suppress the chemiluminescence response of PMNs significantly. This suppression was mediated by the continuous presence of histamine in the culture media. Hydrogen peroxide generated chemiluminescence was similarly suppressed due to high histamine concentrations. Moreover, histamine has a pharmacological role in the control of the oxidative burst reaction of bovine PMNs [98]. The presence of histamine receptors on bovine peripheral blood lymphocytes (PBL) was screened [99], and it was found that bovine PBLs have both H1 and H2 receptors on their surfaces. Moreover, histamine-induced suppression of PBL mitogenesis was mediated only by H2 receptors and not by H1 receptors.

Histamine also plays an important role in normal ovulation, blastocyst implantation, placental blood flow regulation, lactation as well as regulating myometrial activity in response to allergic or infectious stimuli, and the normal parturition process [100,101]. Pregnancy-dependent alterations in histamine concentrations in blood plasma and reproductive tissues have been documented in bovine [102]. Accumulating findings suggest that myometrial cells express histaminergic receptors and cause uterine contraction and relaxation through H1 receptors and H2 receptors, respectively [100,103]. Aberrant uterine contraction may lead to certain pathological states such as preterm labor, dysfunctional labor, and powerful contractions at term leading to fetal hypoxia and distress. H1R was reported in buffalo myometrium and found to have an important role in regulating myometrial contractility during normal and pathological states [104]. Wang *et al*. [105] demonstrated that high expression of H2R and low expression of H1R in the endometrial tissue impaired the inflammatory immune response and prevented excessive uterine tissue damage in metritis cows. Sun *et al*. [106] demonstrated that histamine increased the mRNA expressions and concentrations of inflammatory cytokines, including TNF-α, IL-6, and IL-1β, thereby inducing the inflammatory response of bovine rumen epithelial cells through the NF-κB pathway. Chang *et al*. [107] reported that histamine activates the inflammatory immune response and impairs casein synthesis in mammary gland of dairy cows during subacute ruminal acidosis.

Although histamine is a small molecule of only 17 atoms, it is considered as one of the most important mediators of inflammation and immune reaction. However, its role in the activity of phagocytic cells during inflammatory conditions is still in its infancy. Identifying histamine roles in bovine reproduction and under normal and inflammatory conditions can help us to target this molecule for some other therapeutic purposes in the future.

## Apelin

Apelin is a novel peptide, and the endogenous ligand for the G protein-coupled APJ receptor, an angiotensin-1-like receptor. It has also been classified as one of the recently discovered stress hormones, which was first isolated from the bovine stomach [108,109]. The physiologically active form of apelin is thought to be apelin-36. Apelin and its receptor APJ play a role in the HPA axis responses to various acute stressors [110]. A role for apelin in the regulation of the HPA axis responses to stress is supported through central administration of (Pyr1) apelin-13, which increases the expression of c-fos, a key indicator of neuronal activity, in the paraventricular nucleus [111]. Using APJ knockout mice, APJ has been shown to play a regulatory role in the modulation of the HPA axis responses to short-lasting stressors such as LPS infection (an immune stressor) and insulin-induced hypoglycemia (a metabolic stressor) [110].

Biologically active apelin and its mRNA have been shown to increase during pregnancy and lactation in the mammary gland of rats and usually reach the highest levels around parturition [112]. In cattle, a large amount of apelin was found in colostrum as well as in processed milk [112]. In the bovine mammary gland, apelin and its mRNA significantly increased during gestation, lactation, and reached the highest levels around parturition. Moreover, a large amount of apelin (14-93 pmol/mL) has been noticed in the colostrum, and it has been detectable in commercial bovine milk [112]. Apelin secreted in milk might work to the advantage of neonates by helping them survive through the modulation of their immune responses [113].

Nutritional status can affect apelin in serum and a hypercaloric diet increases adipose tissue and plasma apelin concentrations [114,115]. Norvezh *et al*. [116] demonstrated that alteration in the secretion of apelin-36 in dairy cow during early lactation may represent an endocrine adaptation mechanism during this critical period of lactation. Bhat *et al*. [117] used milk proteome analysis technique to explore the bioactive components of bovine milk and identified apelin as a major regulator of immune system in Kashmiri cattle. Furthermore, apelin appears to have an almost similar anti-inflammatory role as that of cortisol during inflammatory conditions. Studying the presence of apelin receptors on bovine phagocytic cells and its capability in modulating their bactericidal activity during stressful conditions as reported in other species is also needed in bovines.

## Conclusion

Stress in any form, external or internal, greatly influences the productivity and well-being of dairy animals. The HPA axis and SNS system coordinate with the phagocytes and maintain through the release of various hormones, as discussed in this review. However, what are the mechanisms whereby the phagocytes and stress hormones interact with each other and what are the critical limits of a particular hormone above which it is detrimental to the welfare of the dairy cows is poorly understood. Therefore, more scientific studies that elaborate the immune-modulating mechanisms of these hormones and their interplay with other physiological pathways may help to select strategies to uncouple immunity from production. Further, genetic selection of stress-resistant animals, optimum nutrition, and regular monitoring of animals’ health during periods of critical stress may help to reduce stress in dairy animals.

## Authors’ Contributions

MNA and AKD have contributed equally to this review.
